# Role of RNAIII in Resistance to Antibiotics and Antimicrobial Agents in *Staphylococcus epidermidis* Biofilms

**DOI:** 10.3390/ijms231911094

**Published:** 2022-09-21

**Authors:** Andrej Minich, Veronika Lišková, Ľubica Kormanová, Ján Krahulec, Júlia Šarkanová, Mária Mikulášová, Zdenko Levarski, Stanislav Stuchlík

**Affiliations:** 1 Faculty of Natural Sciences, Comenius University in Bratislava, Ilkovičova 6, 841 04 Bratislava, Slovakia; 2 Biomedical Research Center, Institute of Clinical and Translational Research, Slovak Academy of Sciences, Dubravska Cesta 9, 845 05 Bratislava, Slovakia; 3 Science Park, Comenius University in Bratislava, Ilkovičova 8, 841 04 Bratislava, Slovakia

**Keywords:** biofilm, oxacillin, phenol soluble modulins, vanillin

## Abstract

*Staphylococcus epidermidis* is a known opportunistic pathogen and is one of the leading causes of chronic biofilm-associated infections. Biofilm formation is considered as a main strategy to resist antibiotic treatment and help bacteria escape from the human immune system. Understanding the complex mechanisms in biofilm formation can help find new ways to treat resistant strains and lower the prevalence of nosocomial infections. In order to examine the role of RNAIII regulated by the *agr* quorum sensing system and to what extent it influences biofilm resistance to antimicrobial agents, deletion mutant *S. epidermidis* RP62a-ΔRNAIII deficient in repressor domains with a re-maining functional *hld* gene was created. A deletion strain was used to examine the influence of oxacillin in combination with vanillin on biofilm resistance and cell survival was determined. Utilizing real-time qPCR, confocal laser scanning microscopy (CLSM), and crystal violet staining analyses, we found that the RNAIII-independent controlled phenol soluble modulins (PSMs) and RNAIII effector molecule have a significant role in biofilm resistance to antibiotics and phenolic compounds, and it protects the integrity of biofilms. Moreover, a combination of antibiotic and antimicrobial agents can induce methicillin-resistant *S. epidermidis* biofilm formation and can lead to exceedingly difficult medical treatment.

## 1. Introduction

The formation of biofilms is the key survival strategy of *Staphylococcus epidermidis* in the host environment [1]. Biofilm is a multicellular bacterial community defined as a consortium of bacteria coated with extracellular polymeric substances (EPSs) composed of polysaccharides, proteins, and eDNA (extracellular DNA) [2]. EPSs and the heterogeneity of the biofilm are the main factors in the development of antimicrobial-resistant strains such as methicillin-resistant strains of *S. epidermidis* (MRSE) [3]. In biofilms, poor antibiotic penetration, slow growth, nutrient limitation, and the formation of persister cells are hypothesized to be responsible for drug resistance.

Several mechanisms have been described in *S. epidermidis* that can recognize and overcome the physical and chemical protection of the host. The most important mechanisms include communication systems such as quorum sensing (QS) regulating multiple virulence factors and biofilm formation [4]. The *S. epidermidis agr* QS contributes to virulence in model infections associated with the biofilm, including endocarditis [5] and osteomyelitis [6], although the exact role of the *agr* QS system differs from the infection development and site of infection. The model *agr* locus represented by *S. aureus* consists of two distinct operons driven by the P2 and P3 promoters [7]. P3 directs the transcription of RNAIII, the effector molecule of the *agr* locus from which the δ-hemolysin encoded by the *hld* gene is translated [8].

In *S. epidermidis*, RNAIII is a 510-nt-long intracellular effector molecule responsible for regulating the expression of many virulence genes [9]. The main principle of RNAIII-dependent regulation of targeted genes expression is the formation of RNAIII duplexes with 5′ un-translated regions (5′UTRs) of targeted genes [10]. By this mechanism, RNAIII mainly inhibits the production of a series of predominantly surface protein A, coagulase (Coa), responsible for maintaining a compact mature form of EPS, adhesins, and in *S. aureus*, for producing transcription factor repressor (rot) of virulent factors [11]. In addition, RNAIII-dependent regulation leads to an increased production of lipases (Geh) and proteases (SspA, SspB) [12].

In an RNAIII-independent manner, AgrA directly upregulates the transcription of *psm* genes at the *psmα* and *psmβ* operons by binding to the appropriate promoter sequences [13]. Phenol-soluble modulins (PSMs) belong to the family of staphylococcal peptide toxins. In *S. aureus*, PSMα show cytolytic activity against human neutrophils and erythrocytes. Overall modulins affect cell motility and act as antimicrobials against specific bacterial species such as *Streptococcus* [14]. *S. epidermidis* has lower cytotoxic activity than *S. aureus* and preferably produces β-type PSMs, suggesting that this species begins its defense earlier in the onset of an infection [15].

Moreover, biofilm-forming strains can be more resistant to antibiotics than planktonic cells, since the matrix of biofilm structures acts as a diffusion barrier [16] due to biofilm heterogeneity and different phenotypes such as the expression of efflux pumps and persister cells inside the biofilm [17].

Since RNAIII is the main effector molecule of the *agr* QS system, inhibition of RNAIII may, therefore, be an effective method for a reduction in the production of toxins and other virulence factors [18]. In recent years, natural antimicrobial compounds have seemed to be suitable anti-QS and antibiofilm agents and many experiments determined the antimicrobial potential of many natural compounds against biofilm formation. Phenolic compounds have been shown to be effective in inhibiting the formation of biofilms and cell growth in pathogenic bacteria [19]. Moreover, these agents have shown modulatory effects on antibiotic resistance in *S. aureus* and *S. epidermidis* by inhibiting the function of efflux pumps, which are one of the important factors in biofilm maturation [20].

In this study, the effect of the previously studied vanillin [21] in combination with oxacillin was examined to investigate the possibility of a synergistic effect in treatment. Next, analyzing the role of RNAIII in biofilm resistance to antibiotics and phenolic compounds, an *S. epidermidis* RP62a-ΔRNAIII deletion mutant with deleted repressor domains was prepared. The deletion strain was used to determine any change in the susceptibility to oxacillin and vanillin and their combination. Moreover, the change in the phenotype was analyzed by implementing CLSM and Congo red agar.

## 2. Results

### 2.1. Biofilm Formation in Presence of Oxacillin and Phenolic Compounds

**Figure 1:**
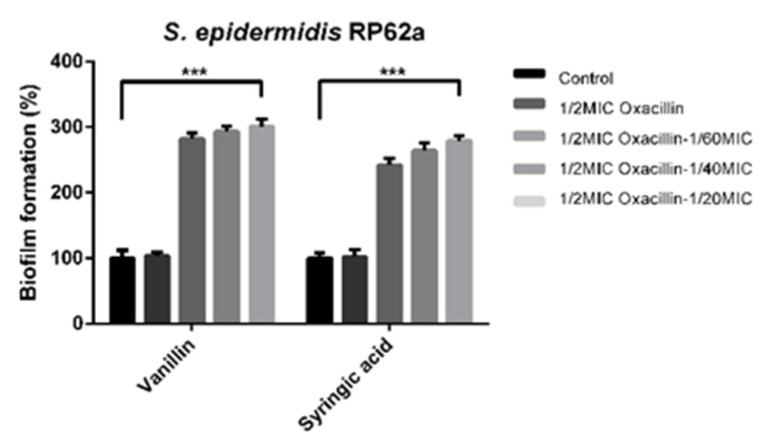
The effect of vanillin and syringic acid in combination with oxacillin on biofilm formation of *S. epidermidis* RP62a (*** *p* < 0.001).

In our previous study [21], vanillin and syringic acid showed a significant antibiofilm and anti-QS effect against *S. epidermidis* biofilms. The next step was to examine whether the selected phenolic compounds can potentiate the antibiotic susceptibility of methicillin-resistant *S. epidermidis*. The addition of oxacillin in a 1/2MIC (MIC 4 mg/mL) concentration determined for the *S. epidermidis* clinical strain 108 was used in combination with vanillin and syringic acid and showed significant induction of biofilm formation (Figure 1).

Vanillin and syringic acid in combination with oxacillin in the determined sub-inhibitory concentrations enhanced biofilm formation. All three concentrations showed that the dose-dependent biofilm induction increased by up to threefold for both phenolic compounds compared to the control. The addition of oxacillin had no effect on biofilm formation, as expected. The same effect of biofilm induction was determined for two more methicillin-resistant clinical strains 745 and 817 with different biofilm genotypes (see the Supplementary Materials S2), which were also studied previously in [21].

To identify to what extent the *agr* QS system and the RNAIII effector molecule potentiate biofilm formation induction and resistance to antimicrobial agents, a deletion mutant *S. epidermidis* RP62a-ΔRNAIII was prepared. This specific genetic manipulation resulted in the deletion of repressor domains with a remaining functional *hld* gene.

### 2.2. Construction of S. epidermidis RP62a-ΔRNAIII

**Figure 2:**
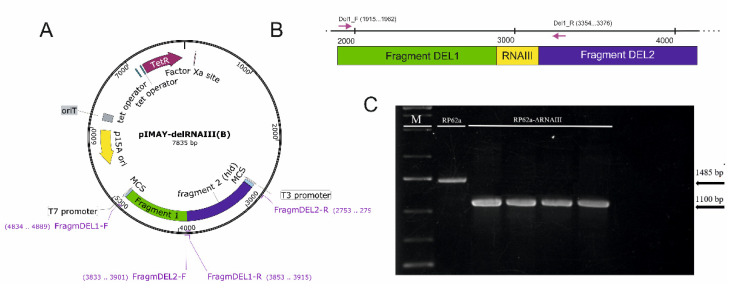
Preparation of *S. epidermidis* RP62a-ΔRNAIII. (**A**). Map of the pIMAY-delRNAIII plasmid used for allelic exchange containing deletion cassette. (**B**). Map of RP62a chromosomal DNA with the RNAIII deleted fragment of the repressor domains sequence with primers used for PCR confirmation of allelic exchange highlighted (green–downstream fragment, blue- upstream fragment, yellow–deleted sequence). (**C**). Electrophoresis gel of confirmation PCR of allelic exchange (1100 bp). As a control, chromosomal DNA of RP62a was used (1485 bp).

To achieve an allelic exchange and deletion of the part of the RNAIII effector molecule without *hld* gene deletion, a plasmid pIMAY-delRNAIII was constructed (Figure 2A) containing a deletion cassette created from two fragments downstream (green) and upstream (blue) from RNAIII (all the details are listed in Supplementary Material S1) (Figure 2B). The plasmid was transformed into *S. epidermidis* RP62a. Transformation and allelic exchange was accomplished using the protocol for staphylococci by Monk et al. [22]. The deletion of the RNAIII fragment was verified by PCR using the primers DEL1-F and DEL1-R, resulting in a length change of 375 bp compared to wild-type RP62a (Figure 2C).

Since deletion of the *agr* locus is frequent when deletion around the *agr* locus is performed, retained *agr* genes were verified by PCR (see Supplementary Material S1) and possible spontaneous mutations in the deletion mutant *S. epidermidis* RP62a-ΔRNAIII were ruled out by sequencing.

### 2.3. Effect of Vanillin and Oxacillin on S. epidermidis Biofilm

**Figure 3:**
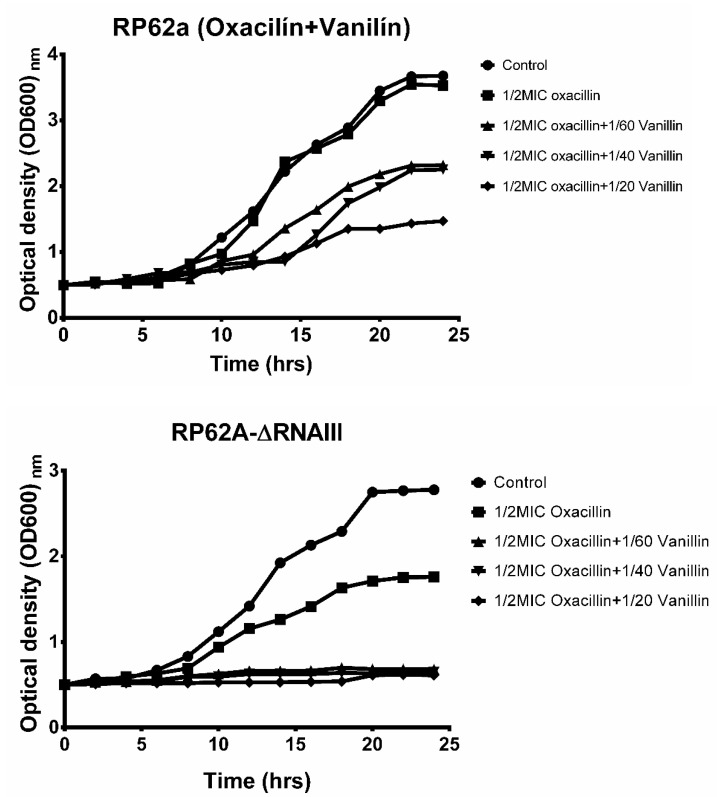
Effect of oxacillin in combination with vanillin on the growth of the methicillin-resistant strain *S. epidermidis* RP62a and the deletion mutant *S. epidermidis* RP62a-ΔRNAIII.

Vanillin was selected to analyze the effect of the phenolic compound in combination with oxacillin on biofilm formation and resistance. First, the 24 h growth curve was determined for both strains (Figure 3). This growth curve for RP62a shows that an addition of 1/2MIC oxacillin had no effect on the growth during 24 h compared to the control. All three sub inhibitory concentrations of vanillin used showed a dose-dependent reduction in bacterial growth. The combination of the highest concentrations of vanillin and oxacillin led to a decrease in the optical density of 67%.

In the case of RP62a-ΔRNAIII, the addition of 1/2MIC oxacillin had a higher inhibition effect and lowered bacterial growth by 46% after 24 h. Moreover, the presence of vanillin and oxacillin together resulted in inhibition of bacterial growth. These results were similar to the methicillin-susceptible strain *S. epidermidis* 108 (see Supplementary Material S2).

**Figure 4:**
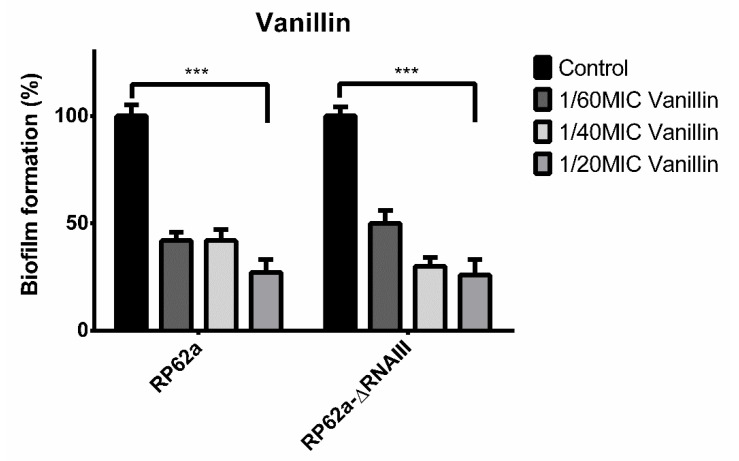
Inhibition effect of vanillin on the biofilm formation of S. *epidermidis* RP62a and RP62a-ΔRNAIII in all three used sub-inhibitory concentrations compared to the non-treated control (*** *p* < 0.001).

Next, the biofilm treatment of both strains using only vanillin was analyzed since biofilm as a structure is more resistant to treatment than planktonic cells (Figure 4). The highest concentration of 1/20MIC vanillin used lowered the biofilm formation by 27% compared to the control. The concentrations of 1/40MIC and 1/60MIC also showed similar effects and inhibited biofilm formation by 50% or up to 70%.

**Figure 5:**
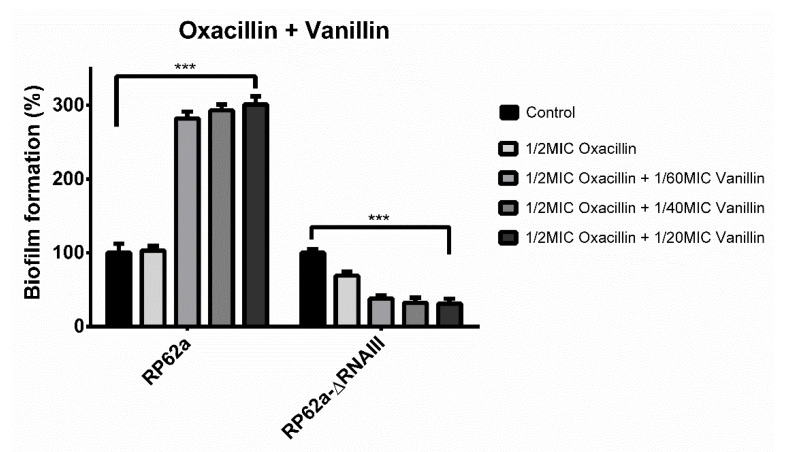
Effect of vanillin in different sub-inhibitory concentrations in combination with 1/2MIC oxacillin on the biofilm formation of *S. epidermidis* RP62a and RP62a-ΔRNAIII. As the results show, wild-type biofilm formation was induced up to threefold compared to RP62a-ΔRNAIII where biofilm was inhibited (*** *p* < 0.001).

However, different results were obtained for the analyzed strains when oxacillin in combination with vanillin was used to treat biofilm (Figure 5). As mentioned, vanillin and oxacillin at the 1/2MIC concentration induced biofilm formation in RP62a of up to 300% compared to the control.

On the contrary, when RP62a-ΔRNAIII was treated with oxacillin in combination with vanillin, inhibition of biofilm formation was observed. Moreover, the use of 1/2MIC only oxacillin lowered the biofilm mass by 37%. The combination of both agents decreased the biofilm formation and the highest concentration of vanillin used allowed the formation of only 27% of the biofilm mass compared to the control.

### 2.4. Real-Time qPCR Analysis of Biofilm-Associated Genes

To obtain a better insight into changes in the gene expression levels, biofilm formation tests using crystal violet staining were supported by relative mRNA expression analysis of biofilm genetic determinants (*icaA* and *aap*), two representative genes of *agr* QS system (*agrA* and *agrD*), and RNAIII-independent genes (*psmβ1* and *psmβ2*).

**Figure 6:**
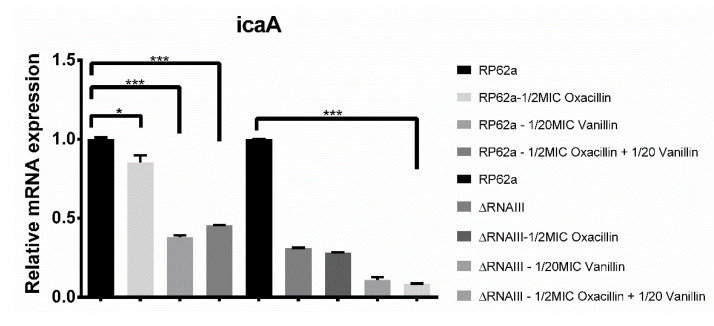

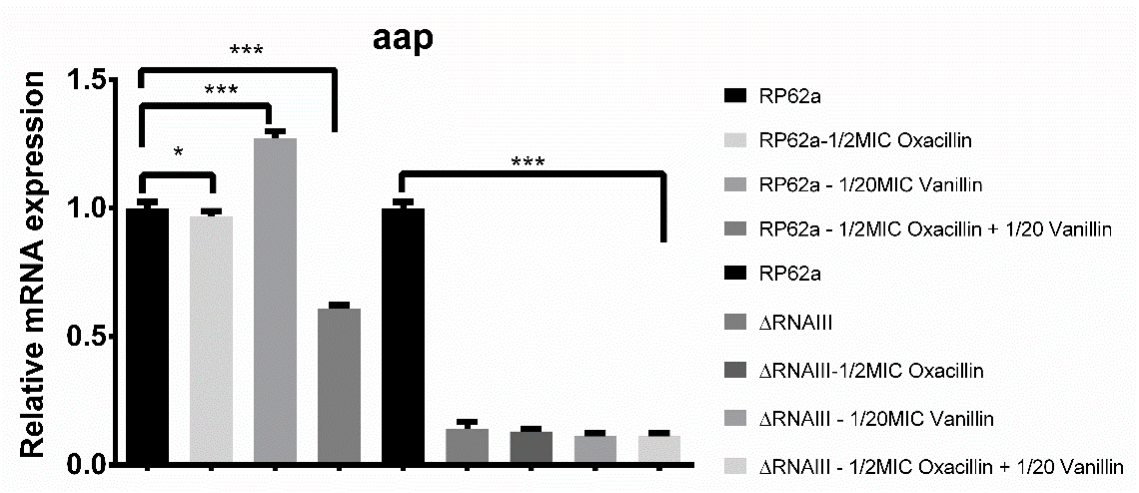
Real-time qPCR analysis of RNAIII biofilm genes *aap* and *icaA*. For both genes, relative mRNA expression was decreased compared to the control. As a control, a housekeeping gene for 16S rRNA and unaffected culture were used (* *p* < 0.01, *** *p* < 0.0001).

Calculation of the relative mRNA expression of the biofilm determinants *aap* and *icaA* genes from RP62A and RP62a-ΔRNAIII in different conditions showed almost identical results (Figure 6). When the 1/2MIC concentration of oxacillin was added to a RP62a biofilm culture, the change in relative mRNA expression was insignificant.

Different results were obtained when treated only with vanillin. *Aap* expression increased by 30% and *icaA* gene expression decreased by 66%. In the presence of oxacillin and vanillin, the same trend in expression was observed and an increase of 40% for the *aap* gene and 55% for the *icaA* gene were detected.

For RP62a-ΔRNAIII, similar changes in relative mRNA expression were obtained. Compared to the wild type, the expression of *aap* and *icaA* decreased by 4- to 5-fold when only oxacillin was added. Approximately, a 70% decrease was also observed for the deletion mutant without the addition of any agent. Equal changes in the relative mRNA expression were also calculated for the other analyzed conditions, leading to a reduction of up to 84% depending on the agent.

**Figure 7:**
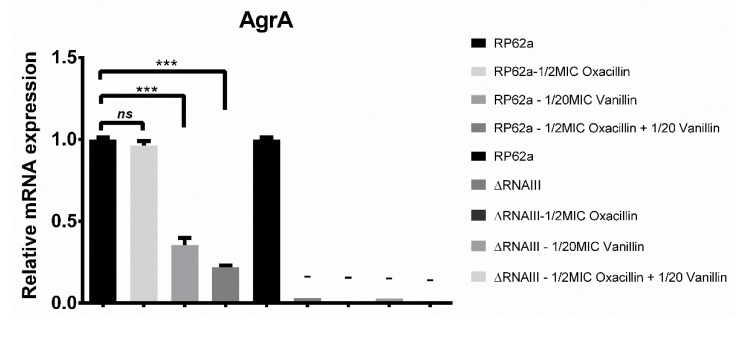

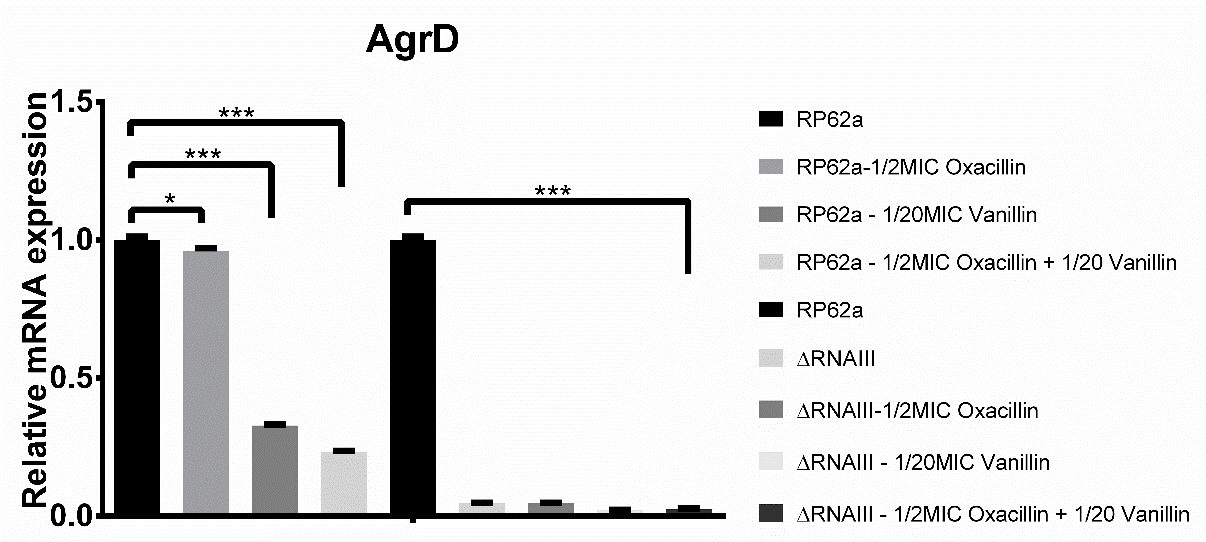
Real-time qPCR analysis of RNAIII-dependent genes *agrD* and *agrA*. For both genes, relative mRNA expression was lowered compared to control. As a control, a housekeeping gene for 16S rRNA and unaffected culture were used (ns—non-significant, * *p* < 0.01, *** *p* < 0.0001).

When the *agr* QS genes were analyzed (*agrA* and *agrD*), similar results for both genes were obtained (Figure 7). Comparing both strains, for the deletion mutant, no expression was detected for *agrA*. For *agrD*, in all conditions, expression was inhibited by up to 99% or no expression was observed. Relative mRNA expression changes for RP62a showed the same trend as for *icaA*. Oxacillin did not affect the expression, the addition of vanillin led to a decrease in the expression from 30% to 60% and the combination of both agents showed an even more pronounced decrease in the expression of up to 70%.

**Figure 8:**
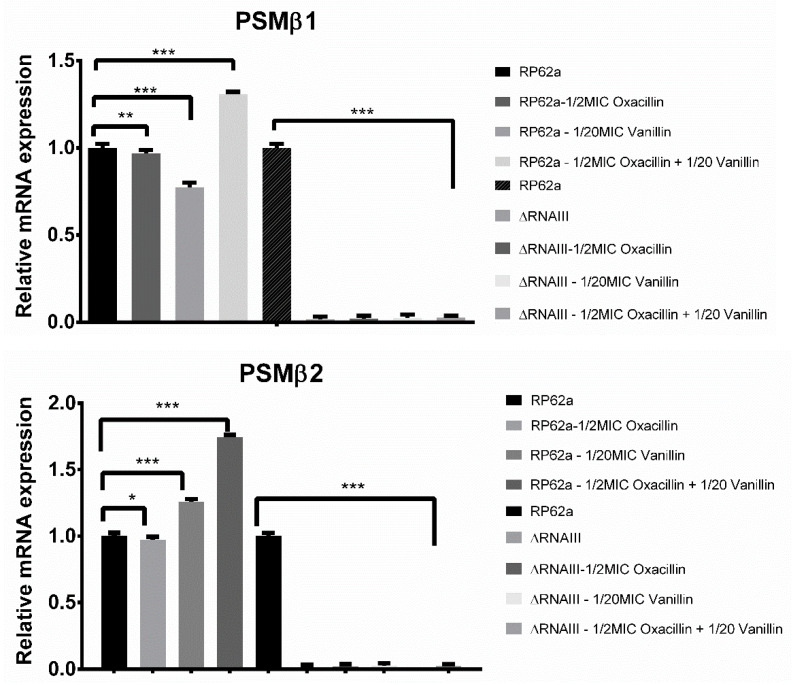
Real-time qPCR analysis of RNAIII-independent genes psmβ1 and psmβ2. For both genes, relative mRNA expression was decreased compared to the control. As a control, a housekeeping gene for 16S rRNA and an unaffected culture were used (* *p* < 0.01, ** *p* < 0.001, *** *p* < 0.0001).

To complete the analysis, the expression of RNAIII-independent genes *psmβ1* and *psmβ2* was determined (Figure 8). For both *psmβ1* and *psmβ2*, in the case of RP62a-ΔRNAIII, relative mRNA expression was decreased by up to 99% or no expression was determined in all analyzed conditions, with no difference related to the agents used.

These results were opposite to the data obtained for RP62a. As for *agrA* and *agrD* or the biofilm determinant genes *aap* and *icaA*, in the presence of oxacillin, no change was observed. Vanillin increased expression by 30%. The highest change was determined in the presence of oxacillin and vanillin combined, resulting in a 35% increase in *psmβ1* and 80% in *psmβ2*.

### 2.5. Analysis of Change in the Phenotype of the RNAIII-Deficient Strain

**Figure 9:**
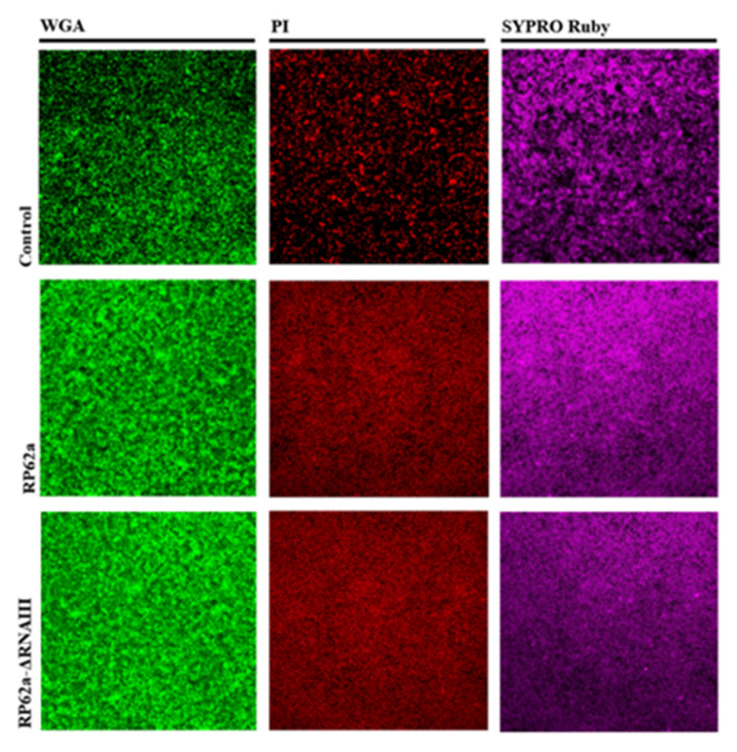
CLSM analysis of EPS treated with oxacillin and vanillin in *S. epidermidis* RP62a and RP62a-ΔRNAIII (magnification 60×). Polysaccharides were stained with WGA; proteins were stained with SYPRO Ruby Biofilm Tracer and eDNA was stained with PI.

**Figure 10:**
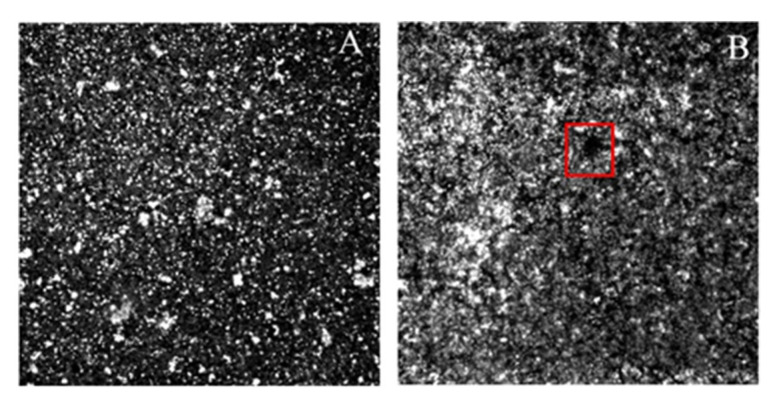
CLSM analysis (60× magnification) of change in the biofilm composition between *S. epidermidis* RP62a (**A**) and RP62a-ΔRNAIII (**B**) (red box shows perforation of biofilm described in results).

**Figure 11:**
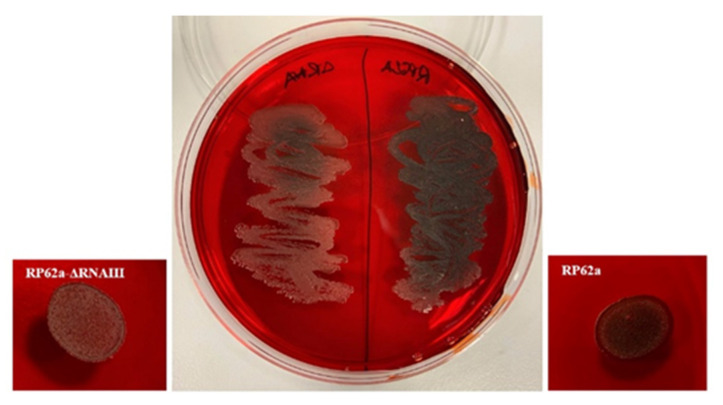
Verification of decreased polysaccharide production in EPS of RP62a-ΔRNAIII (left) compared to RP62a (right) on Congo Red agar. Figure also compares change in results when culture is dropped and spread (center Figure).

In order to complete the analyses of differences in genotypes, a comparison of strains by CLSM and Congo Red agar was carried out to determine any phenotypic differences (Figure 9, Figure 10 and Figure 11). Both methods agree with the results described in Section 2.3 and Section 2.4. CLSM confirmed the enhancement of the production of polysaccharides in strain RP62a compared to the control (Figure 9) when the strains were treated with oxacillin and vanillin. The same trend was observed for proteins and eDNA. RP62a-ΔRNAIII showed the same results and higher signal, suggesting overexpression of the EPS components. Even the real-time qPCR and microtiter-plate analyses showed an inhibition effect.

CLSM revealed a change in the structure of the biofilm of RP62a-ΔRNAIII (Figure 10). The strain deficient in the RNAIII gene showed a different composition of EPS (Figure 10, marked by red square) and contained perforated structures throughout the biofilm compared to the wild-type strain (this microscopic picture was selected for representation of perforations).

To support the findings of the CLSM, Congo Red agar was used (Figure 11) to determine differences in biofilm formation. A drop of an overnight culture of both strains and the bacterial streak showed differences in their phenotypes. RP62a produced more polysaccharides and appeared black on Congo Red agar. Oppositely, the deletion strain RP62a-ΔRNAIII appeared red after 24 h, showing lower polysaccharide production and supporting the real-time qPCR and microtiter plate analyses.

## 3. Discussion

Due to the unique properties of biofilm formation, according to the WHO, *S. epidermidis* causes up to 80% of nosocomial infections [23,24]. Based on their capability to colonize various living and non-living surfaces, up to 31% of diseases are associated with bloodstream infections, especially after the introduction of central venous catheters, urea catheters, artificial joint replacements, and heart valves [25]. Phenolic compounds have potential to be suitable antimicrobial agents since an antimicrobial effect on the planktonic stages of bacterial cells and inhibition of mature cells was observed in biofilms without an antibacterial effect, especially in some pathogens such as *Listeria monocytogenes* [26], *Pseudomonas aeruginosa* [27], *Streptococcus mutans* [28], *S. aureus*, and *S. epidermidis* [29].

In our previous study, vanillin and syringic acid were selected as antimicrobial agents and their antibiofilm and anti-QS properties were determined [21]. In the present study, vanillin was used to study the potential enhancement of susceptibility to antibiotics, in this case, oxacillin, since similar results of enhancement were obtained for biofilms of *S. aureus* [30], *Klebsiella pneumoniae* [31], and *E. coli* [32]. As an example, previously analyzed flavonoids (−)-epicatechin gallate and (−)-epigallocatechin gallate diminished β-lactam MICs to the antibiotic breakpoint in *S. aureus* [33,34]. The same effect of the combination of an antimicrobial agent with an antibiotic was determined for patulin combined with tobramycin, resulting in effective killing of bacterial cells [35].

To determine if vanillin or syringic acid can potentiate the antibiotic susceptibility of methicillin-resistant *S. epidermidis*, oxacillin at a concentration of 1/2MIC (see the material and methods) determined for the susceptible strain *S. epidermidis* 108 was used with the same concentrations of vanillin and syringic acid (concentrations in the materials and methods) from a previous study [21] (Figure 1). The addition of 1/2MIC of oxacillin and vanillin or syringic acid significantly enhanced the biofilm formation at all used sub-inhibition concentrations of phenolic compounds by up to 300% compared to the control. Both combinations did not influence the culture treated only with oxacillin. The same effect of induction was observed for *P. aeruginosa* and *Agrobacter tumefaciens*, where vanillin and the other examined phenolic compounds alone increased the capacity of biofilm formation by 2- to 7-fold depending on the agent used [36]. However, induction was observed only for the use of antimicrobial agent, not in combination with antibiotic.

To assess the influence of RNAIII repressor domains on the induction of biofilm formation and resistance to antimicrobial agents, RNAIII-deficient mutant *S. epidermidis* RP62-ΔRNAIII was prepared with the functional *hld* gene (Figure 2). Similar mutants of *S. epidermidis* were prepared, in which the whole *agr* QS locus was deleted [13]. In this study, only part of the RNAIII coding repressor domains was deleted. An allelic exchange cassette was constructed by amplification of downstream and upstream fragments from the targeted sequence and fused to the functional cassette. (Figure 2A). Successful allelic replacement was then verified by PCR using selected primers (Figure 2B), resulting in a length change of 385 bp (Figure 2C) compared to the wild-type RP62a. Since agr locus deletion can occur frequently, confirmation PCR analysis of the *agrA*, *agrD*, and *hld* genes was performed for wild-type and deletion mutant strains (Figure S1 in Supplementary Material S1). Moreover, parts of the *agr* locus (*agrB*, *agrD*, and *agrC*, part of *agrA*) were sequenced and no difference was determined.

Next, only the effect of vanillin was examined since the results from our previous study [21] were comparable to those of syringic acid. The growth curves of cultures that were untreated, treated with oxacillin, and treated with oxacillin and vanillin were determined (Figure 3). To determine the growth curves of the analyzed strains, BHI medium was used instead of TSB supplemented with 1% glucose since media with a high content of NaCl or glucose induces biofilm formation [37,38]. The curves of the 24 h untreated cultures showed relatively minor changes in the optical density (OD_600_). For RP62a-ΔRNAIII (OD_600_ 2.34), OD_600_ decreased by 13% compared to wild-type RP62a (OD_600_ 2.71). A more significant difference was observed for the treatment with the 1/2MIC oxacillin concentration, where OD_600_ of the RNAIII-deficient mutant decreased by 45% compared to the control and wild-type RP62a. The same effect of oxacillin was observed for the methicillin-susceptible strain *S. epidermidis* 108 (see Supplementary Material S2). A combination of 1/2MIC of oxacillin and the analyzed vanillin inhibited the bacterial growth of RP62a-ΔRNAIII completely. The same effect of inhibition was determined only for high doses of antibiotics for infected cardiovascular-implantable electronic device (CIEDs) pathogens *P. aeruginosa*, *S. aureus*, and *E. coli*, which were resistant to antibiotics used for treatment [39].

Since biofilms are 10 to 1000 times more resistant to common antibiotics than their planktonic state [2], the effect of vanillin alone or in combination with 1/2MIC oxacillin on biofilm formation was studied (Figure 4 and Figure 5). As data show (Figure 4), vanillin alone at all sub-inhibitory concentrations showed the same trend of inhibition of biofilm formation for both strains. The highest used sub-inhibitory concentration of vanillin (1/20MIC) lowered biofilm formation by up to 27% compared to the control (Figure 4). Different results were obtained when a combination of antibiotic and phenolic compound was used. As mentioned before, biofilm formation of RP62a increased by up to 300%. On the contrary, when RP62a-ΔRNAIII was treated with antibiotic in combination with vanillin, an inhibitory effect of biofilm formation was observed, allowing the formation of only 27% of the biofilm mass (Figure 5). Moreover, the use of 1/2MIC oxacillin only decreased the biofilm mass by 37%. Some studies have demonstrated that low doses of antibiotics can induce biofilm formation, indicating that biofilm regulation includes the presence of antibiotics. However, this phenomenon is currently unclear and remains under investigation [40,41]. These findings also suggest that repressor domains of RNAIII can regulate other genes as described in Gupta et al. [42]. This study showed RNAIII regulation of mgrA through binding to two regulation sites of mgrA, one of which was deleted in this study. Moreover, the study of Gupta et al. showed no effect on resistance to oxacillin. The combination of vanillin and oxacillin, which affects the survival of viable cells, could lead to stress enhancement and the involvement of unknown factors, enhancing biofilm formation

In this study, the results also showed significant enhancement of the susceptibility to oxacillin. These findings suggest a significant impact of the RNAIII repressor domains on the regulation of other virulence factors through transcriptional regulators such as mgrA; however, more research in this direction is needed. These results also show that biofilm EPS provides high defense against antimicrobial agents and antibiotics [13].

To support the microtiter plate method findings, real-time qPCR analysis was performed. Calculation of the relative mRNA expression of the biofilm determinants *aap* and *icaA* genes from RP62A and RP62a-ΔRNAIII in different conditions showed almost identical results (Figure 6). Oxacillin at the 1/2MIC concentration changed the relative mRNA expression. Regarding the biofilm cultures treated with only vanillin, *aap* expression increased by 30% and *icaA* gene expression decreased by more than 60%. In the presence of oxacillin and vanillin, *aap* expression decreased by 40% and 55% for the *icaA* gene.

For RP62a-ΔRNAIII, compared to the wild type, the expression of *aap* and *icaA* decreased only when oxacillin was added, resulting in a 4- to 5-fold decrease. The absence of both agents had a similar effect and equal change in the relative mRNA expression, which was calculated from cultures in the presence of both agents, of up to 84% (Figure 6).

When *agr* QS genes were analyzed (*agrA* and *agrD*), comparable results for both genes were obtained for the RP62a strain (Figure 7). When the expression of *agr* genes influenced by the analyzed antimicrobial agent or oxacillin decreased, biofilm formation increased. These results agree with a previous finding, where *agr* deficiency or lower expression enhanced biofilm formation [43]. Comparing both strains, for the deletion mutant, no expression was detected for *agrA*. For *agrD*, in all conditions, expression was inhibited by up to 99% or no expression was observed. Interestingly, even the absence of *agrA* and *agrD* expression in all conditions for the RNAIII-deficient mutant had no influence on biofilm formation and this strain formed only 10% less biofilm mass compared to the wild-type strain RP62a. The same effect was observed for the deletion of the *agrD* gene in *S. aureus*, where no change, or even enhancement, in biofilm formation was observed [44]. A partial explanation could be that under certain conditions, quorum sensing may play a role in biofilm development, inducing genes responsible for the attachment to the surface but may not play an obvious role in biofilm development under all conditions [43]. The *agr* QS system is thought to exercise regulatory control over a wide range of staphylococcal genes such as the analyzed psmβ1 and psmβ2, many lipases, exoproteases, or virulence factors [45]. However, conditions that result in an increase biofilm formation were not determined and remain unknown.

Greater insight into the crucial part of RNAIII repressor domains in biofilm resistance was offered by the analysis of *psmβ1* and *psmβ2* gene expression (Figure 8). As the data shows, relative mRNA expression increased by up to 37% for *psmβ1* and 80% for the *psmβ2* gene when wild-type RP62a was treated with the combination of oxacillin and vanillin compared to the control. These findings are contradictory to the finding published by Queck et al. [13]. This study showed enhanced PSM production even when the expression of agrA-regulating *psm* genes was decreased. On the contrary, no agrA expression led to no expression of PSMs in RP62a-ΔRNAIII. Increased PSM expression in the wild-type strain can be influenced by other non-identified regulator molecules; however, more analyses are needed to further explain the observed effect.

CLSM confirmed the overexpression of PSMs for RP62a (Figure 9), where the almost 2-fold increase detected by qPCR correlated with the enhanced signal of protein in the sample under microscopy. These findings agree with microtiter plate analyses (Figure 3, Figure 4, Figure 5 and Figure 6). Moreover, as CLSM shows, other components were enhanced, such as polysaccharides and eDNA. Interestingly, CLSM showed the same results for RP62a-ΔRNAIII. The signals of all three components were 3 to 4 times higher compared to the control. Even staining with crystal violet showed an inhibitory effect (Figure 6).

One of the hypotheses is the role of PSMs in the biofilm structure. All PSMs of *S. aureus* and *S. epidermidis* PSMβ peptide structure biofilms are so far the only bacterial factors for which roles in the in vivo dissemination of biofilm-associated infection could be directly demonstrated [15,39]. In isogenic *S. aureus*, *psm* gene deletion mutants led to highly disrupted formation of biofilm channels, abolishment of the characteristic waves of biofilm detachment and regrowth, and loss of control of biofilm expansion [46]. The described characteristics of disrupted biofilm channels were determined for the RNAIII-deficient strain RP62a-ΔRNAIII (Figure 10) compared to the wild type. This may explain why the biofilm of the deficient strain showed an increased signal even when the inhibition effect was determined. The hypothesis is that with no PSM expression, biofilm formed but was less rigid and flatter, without the typical “mushroom form” 3D structure and other layers of cells were captured by CLSM (Figure 9).

The Congo Red method was used to supplement the other method and detect any possible changes in the phenotype. The biofilm 24 h culture of RP62a produced more polysaccharides and appeared black. Oppositely, the deletion strain RP62a-ΔRNAIII appeared red after 24 h, displaying lower polysaccharide production (Figure 11).

This study confirms that vanillin alone can act as an antibiofilm and anti-QS agent. When the selected phenolic compounds are combined with antibiotic, in this case, oxacillin, possible stress conditions lead to enhanced expression of PSMs by unknown mechanism, and polysaccharides and eDNA form rough EPS and protect cells inside the biofilm of methicillin-resistant *S. epidermidis*. The RNAIII-deficient strain produced very low or no PSMs became susceptible to oxacillin and with vanillin treatment had synergistic effects and no growth was detected.

These results provide a hypothesis that for *S. epidermidis*-producing biofilms determined as a cause of infection, it is important to determine whether the strain is resistant or susceptible to antibiotics used for treatment since when treated with antimicrobial agents or their combination, it can lead to an increase in biofilm formation, and lead to more difficult treatment of infection.

## 4. Materials and Methods

### 4.1. Construction of S. epidermidis RP62a-ΔRNAIII

Briefly, the fragments DEL1 and DEL2 were amplified by specific primers (the primers are listed in Supplementary Material S1) from genomic DNA of RP62a. Next, both fragments were joined together by fusion PCR using only the outer primers of each fragment and a cassette for allelic exchange was prepared. For PCR, Dream Taq DNA polymerase (Thermo Fisher Scientific, Waltham, MA, USA, cat. no. EP0701) was used. The cassette delRNAIII was cloned into the plasmid pIMAY using the restriction sites *NotI* and *EcoRI* and the plasmid pIMAY-delRNAIII was constructed. The protocol by Monk et al. [20] was used for electroporation and allelic exchange in *S. epidermidis*.

### 4.2. Bacterial Strains and Culture Conditions

The biofilm-producing strain *S. epidermidis* RP62A (ATCC 35984) and the prepared deletion mutant *S. epidermidis* RP62a-ΔRNAIII was examined. Strains were maintained in BHI medium for determination of the growth curve. For the biofilm assays, a TSB medium supplemented with 1% glucose was used to induce biofilm formation. For the selection of positive clones, BHI agar was used as described in the protocol by Monk et al. [22].

### 4.3. Antimicrobial Agents and Antibiotics

Vanillin was isolated from oak bark lignin waste and was provided by the Slovak Technical University in Bratislava. Vanillin was stored at room temperature and diluted at subinhibitory concentrations in 99.9% dimethylsulfoxide (DMSO). The range of used subinhibitory concentrations of vanillin (1/60MIC, 1/40MIC, 1/20MIC) and syringic acid in this work was prepared from stock solution at a concentration of 250 mM/mL. Stocks were stored at 4 °C when used for the assays. The sub-inhibitory 1/2MIC concentration of oxacillin was prepared from stock solution at a concentration of 4 mg/mL.

### 4.4. Assessment of Biofilm Formation by Crystal Violet Staining

The biofilm formation assay was analyzed by crystal violet staining for staphylococci [47]. Overnight cultures were cultivated at 37 °C and were diluted with TSB supplemented with 1% glucose at a 1:100 ratio, and 200 µL of bacterial culture was poured into the well. For the inhibition effect analysis, 20 µL of antimicrobial agent was poured into 180 µL of bacterial culture in the well and biofilm was grown at 37 °C for 24 h. For staining, a solution of 0.1% crystal violet was used. The optical density (OD) was measured at 570 nm using a microtiter-plate reader (Varioskan Flash, Thermo Fisher Scientific, Waltham, MA, USA).

### 4.5. Determination of the Growth Curve of Treated and Untreated Strains

During determination of the bacterial growth rate for 24 h, strains were grown in BHI medium at 37 °C, with shaking at 160 rpm. These conditions were maintained by a microtiter plate reader (Varioskan Flash, Thermo Fisher Scientific, Waltham, MA, USA). The optical density (OD) was measured every hour at 570 nm.

### 4.6. Confocal Laser Scanning Microscopy (CLSM)

WGA (Wheat Germ Agglutinin, Alexa Fluor™ 488 Conjugate, Thermo Fisher Scientific, Waltham, MA, USA, cat.no. W11261) at a final concentration of 5 µg/mL was used to stain PIA/PNAG in EPS, PI at a final concentration of 3 µg/mL was used to stain eDNA, and FilmTracer™ SYPRO™ Ruby Biofilm Matrix Stain (Thermo Fisher Scientific, Waltham, MA, USA, cat.no. F10318) at the final concentration in the protocol was used to stain proteins. Welco wells B.V. (Netherlands) glass-bottom Petri dishes were used to form biofilm. After 24 h, biofilms were washed with PBS, and stained with appropriate dye. Microscopy was performed on a FluoroView FV1200 (Olympus LifeSciences), and images were adjusted and correlated by FluoroView FV1200 Software. An oil objective with 60× zoom was used for imaging.

### 4.7. Congo Red Agar Analysis

To analyze the differences in the phenotypic production of biofilm, the modified Congo Red agar method of Kaiser et al. [48] was used. BHI agar with sucrose (5%), Congo Red (0.08%), NaCl (1.5%), glucose (2%), and vancomycin (0.5 mg/mL) was prepared. A volume of 20 µL of overnight culture was dropped on the agar plate, air-dried, and incubated for 24 h at 37 °C.

### 4.8. Phenol-Chloroform RNA Extraction

The biofilm was grown in a polystyrene Petri dish for 24 h in static conditions at 37 °C and washed with PBS (pH 7.2) to remove planktonic cells. The biofilm was scraped off, nuclease-free water (1 mL) was added to the dish, and cells were transferred to a microcentrifuge tube and pelleted by centrifugation at room temperature at 13,400 rpm (MiniSpin^®^ Eppendorf—rotor F45-12-11) for 5 min. The pellet was resuspended with 100 μL of RNase-free water, 100 μL of a phenol: chloroform mixture at a 1:1 ratio was added, and the mixture was incubated at 70 °C for 30 min with shaking followed by centrifugation. The water fraction was then precipitated with isopropanol. Pellet was washed with 70% ethanol two times, air-dried, and resuspended in nuclease-free water. The concentration of RNA was measured by a NanoDrop (Thermo Fisher Scientific, Waltham, MA, USA).

### 4.9. Real-Time qPCR

A One-Step PCR kit (Qiagen), following the standard manufacturer’s protocol, was used. For all genes, the primer annealing temperature was set to 60 °C, and for amplification, Roche LightCycler^®^ 480 96-well half-skirted plates were used. Real-time analysis was performed in triplicates on the Light Cycler^®^ 480 System by Roche. The primers are listed in the Supplementary Materials (Table S1).

### 4.10. Statistical Analysis

All the measurements were performed in biological triplicates. Values as mean ± SD were obtained from Microsoft Excel, and statistical significance was evaluated by the *t*-test (*p* < 0.05) or one-way ANOVA (*p* < 0.01).

## 5. Conclusions

The presented study demonstrates the effect of oxacillin in combination with vanillin as an antimicrobial agent against methicillin-resistant *S. epidermidis*. For this research, vanillin was selected based on our previous study and potential enhancement of antibiotic susceptibility was determined. To analyze the role of RNAIII in biofilm resistance to antimicrobial agents, the *S. epidermidis* RP62a-ΔRNAIII strain was prepared. Our findings show that treatment of biofilms with oxacillin in combination with vanillin can enhance the biofilm formation of wild-type RP62a by up to threefold compared to the control. The opposite effect of these agents was observed for the RP62a-ΔRNAIII strain, where biofilm formed only up to 27% compared to the control.

These results were supported by real-time qPCR. Data showed lowered relative mRNA expression or no expression compared to the control of all analyzed genes. The CLSM analysis and the Congo Red method supplemented the findings of importance of the RNAIII repressor domains and its important role in the biofilm structure and resistance to antimicrobial agents and antibiotics. When PSMs were not expressed, the biofilm was flatter with a perforated structure in EPS and lowered polysaccharide and protein production led to the formation of more susceptible biofilm to antimicrobial agents and antibiotics. Oppositely, stress conditions triggered by vanillin and oxacillin led to increased biofilm production when treated with oxacillin and vanillin, suggesting the importance of knowing whether the strain is antibiotic resistant or susceptible to select the optimal treatment.

## Data Availability

Not applicable.

## Supplementary Materials

The following supporting information can be downloaded at: https://www.mdpi.com/article/10.3390/ijms231911094/s1. Reference [49] is cited in the supplementary materials.
