# Supplementary material for: Role of RNAIII in Resistance to Antibiotics and Antimicrobial Agents in Staphylococcus epidermidis Biofilms
Source: Int J Mol Sci. 2022 Sep 21;23(19):11094. doi: 10.3390/ijms231911094 (PMC9569910; doi:10.3390/ijms231911094)
Supplement: Supplementary file 1 [file ijms-23-11094-s001.zip › Supplementary material S1.pdf]

PCR amplification of allelic exchange cassette

Table S1. Primers used in this work.

Primer name	Sequence	Length	Reference
rt_aap_F	TGAGGCCGTACCAACAGTG	103 bp	This work
rt_aap_R	ATGGGCAAACGTAGACAAGGT		
rt_icaA_F	TGATCCTACGCACATCGCTT	94 bp	This work
rt_icaA_R	CGAACCACGTGCTCTATGCT		
agrA_F	GTTTGTGAAGATGACCAAAGACA	104 bp	This work
agrA_R	AGGATCATTTGTTGCTAAAGC		
agrD_F	CACTACAATCTTGGAATTTATTGG	89 bp	This work
agrD_R	TCTGGTACTTCTGGTTCGTCAA		
PSMβ1_F	AGCAGCCATCACTAACG	90 bp	This work
PSMβ1_R	CCCAAAAATCGATTCACCATATC		
PSMβ2_F	GATGCAGGAATCAACCAAGATTG	93 bp	This work
PSMβ2_R	GACCTAATAATTTAGAAATAACACTAATACC		
DEL1-F	GGCCGCGGCCGCCAAGAATATTTTTATTTTAATTATTACTTGATAATTAAATGTAAGCTA	1002 bp	This work
DEL1-R	CAGTTAATAAATTCAAAAAATAATTTTAACTATGCTATTTATAAAGATGTGGTTTTAGATTTC		
DEL2-F	CATCTTTATAAATAGCATAGTTAAAATTATTTTTTGAATTTATTAACTGTATCGATAATCCATTTTACT	1120 bp	This work
DEL2-R	GGCCGAATTCTCTAGGGTTATATTTACTCGTATAGTTTAGTCAG		

To assemble both fragments to functional allelic exchange cassette, fragments were assembled together with outer primers. In this case fragment DEL1-F and DEL2-R were fused. Cleavage sites on primers are underlined.

Table S2. Combinations of primers used for amplification of framents and deletion cassette with appropriate lenghts of fragments.

Primer name	Fragment name	Product length
DEL1-F/DEL1-R	Fragment 1	1002 bp
DEL2-F/DEL2-R	Fragment 2	1120 bp
DEL1-F/DEL2-R	Functional allelic exchange cassette	2115 bp

Construction of plasmid pIMAY-delRNAIII

Both, plasmid pIMAY and functional cassette as PCR product were digested by restriction enzymes NotI and EcoRI (New England Biolabs) in a 50 μL reaction. CutSmart buffer was used for restriction and reaction was incubated at 37 ° C for 1 h. Digested vector was dephosphorylated with alkaline phosphatase (rSAP, New England Biolabs) for 30 min at 37 °C. Dephosphorylation reaction was deactivated at 65 °C for 10 min. Both, vector and insert were purified by Wizard®SV Gel and PCR clean-up system (Promega). For ligation, the volume of digested vector and insert were determined based on concentration after digestion. Ligation was performed in 20 μL reaction containing T4 DNA ligase (New England Biolabs) andT4 DNA liagase buffer (supplied by manufacturer) for 1 h at laboratory temperature. 5 μL of liagtion mixture was transformed to *E. coli* strain E. cloni 10G (Merck) and positive clones were selected on chloraphenicol (stock solution 10 mg/ mL).

Transformation of E. coli Ec_SERP62aI to overcome S. epidermidis RM systems

To overcome both RM systems of *S. epidermidis* and increase of transformation effieciency in staphylococci, prepared construct was transformed to *E. coli* strain Ec_SERP62aI for proper methylation (Lee et al., 2019 [45]).

To 50 μl of chemocompetent *E. coli* cells, we added 1μg of plasmid DNA pIMAY-delRNAIII. Competent cells together with the plasmid DNA were incubated on ice for 10 min. Subsequently, we exposed the cells to a heat shock of 42 ° C for 45 sec. After heat shock, the cells were incubated again on ice for 2 min. 0.5 ml of liquid LB medium was added to the cells, mixed, transferred to a microtube and incubated with shaking at 37 ° C for 1 hour. The cells were then plated on Petri dishes containing LB medium with 10 μg/ mL chloramphenicol and incubated at 37 ° C overnight. Selected colonies were verified for plasmid DNA.

Electroporation of S. epidermidis and allelic exchange

Electroporation was conducted essentially as described by Löfblom et al. (2007) [1] and allelic exchange as described by Monk et al. (2012) [22].

Verification of presence of agr locus genes

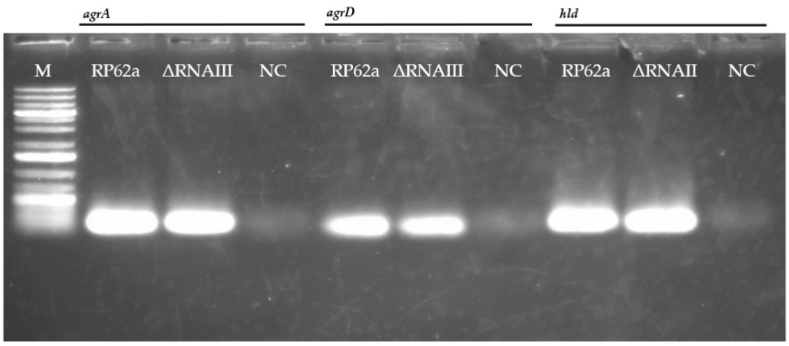

Figure S1.: Verification of presence of agr locus genes. Three genes *agrA*, *agrD* and *hld* were verified by PCR using primers used in Real Time qPCR (Table 1) (NC – negative control).

Reference

1. Löfblom J., Kronqvist N., Uhlén M., Ståhl S., Wernérus H. Optimization of electroporation-mediated transformation: *Staphylococcus carnosus* as model organism. *J Appl Microbiol.* **2007**, Mar; 102(3): 736-747. doi: 10.1111/j.1365-2672.2006.03127.x.
