# Supplementary material for: Role of RNAIII in Resistance to Antibiotics and Antimicrobial Agents in Staphylococcus epidermidis Biofilms
Source: Int J Mol Sci. 2022 Sep 21;23(19):11094. doi: 10.3390/ijms231911094 (PMC9569910; doi:10.3390/ijms231911094)
Supplement: Supplementary file 1 [file ijms-23-11094-s001.zip › Supplementary material S2.pdf]

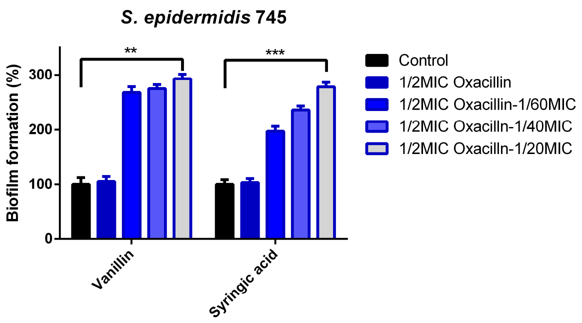

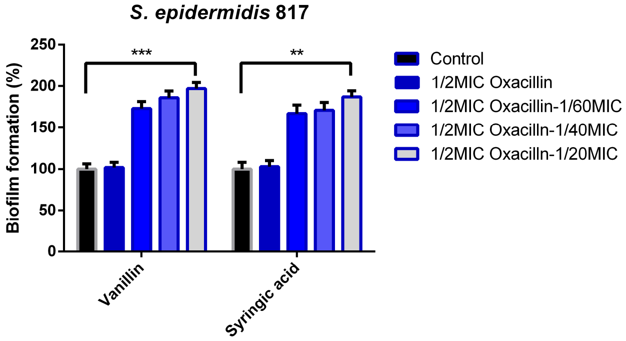

Figure S1: Effect of vanillin and syringic acid in combination with oxacillin on biofilm formation on *S. epidermidis* 745 and 817 (** p <0.01, *** p <0.001).

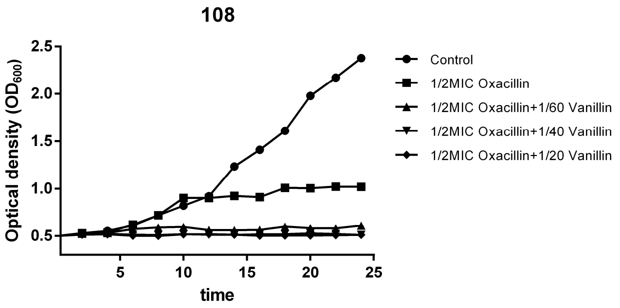

Figure S2: Analysis of the effect of Oxacillin in combination with vanillin on the growth of methicillin susceptible clinical strain *S. epidermidis* 108.
